# Exploring hearing loss, cognition and MRI measures of hippocampal substructure in adults

**DOI:** 10.1002/alz70856_103991

**Published:** 2025-12-24

**Authors:** Imola X MacPhee, John AE Anderson

**Affiliations:** ^1^ Carleton University, Ottawa, ON, Canada

## Abstract

**Background:**

Hearing loss and impaired hearing in noise can lead to macrostructural (i.e. volume and thickness) gray matter changes in the hippocampus linked to cognitive decline. Critically, the hippocampus contains several subfield regions with unique functional properties. However, little is known of sub‐region function in the context of hearing in humans. In animals, the Cornus Ammonis 1 (CA1) is tonotopically organized and may be affected by frequency‐specific impairment. In humans, CA1 is associated with verbal memory and CA4 has been associated with free recall. Here, we use HippUnfold, a machine‐learning based automated hippocampal unfolding and subfield segmentation technique, to better understand the relation between hearing and cognition in the brain.

**Method:**

Preliminary results include 26 young (18‐30 yrs) and 19 older (60+ yrs) cognitively healthy adults from Ottawa, Canada. Assessments included hearing (pure tone audiometry, QuickSIN), cognition (MoCA and Shipley‐2), and 3T MRI (T1 images). Multiple linear regression was used to assess total hippocampal volume. Multiple Factor Analysis (MFA), a multivariate statistical technique, was used to simultaneously reduce dimensionality, analyze the data in hierarchical blocks and generate a set of principal components called dimensions, that relate brain and behaviour elements.

**Result:**

Total hippocampal volume decreased as expected with increasing age and hearing difficulty, (*F*(2, 42) = 5.97, *p* =  0.005). Dimension one of the MFA explained 23% of the variance and was associated with features of the subiculum, CA1 and CA4, with near‐equal contributions from age, hearing in noise (QuickSIN), and cognition (Shipley blocks). In the second dimension, age loaded most strongly along with features of CA3 (11% of the variance). Exploratory analyses with hearing thresholds in older adults showed dimension one driven by age (25% of the varIance), and dimension two driven by cognition (MoCA), left ear hearing thresholds, sex and left hippocampal subfield measures (18% of the variance).

**Conclusion:**

Our findings identified, for the first time, an association between the subiculum, CA4 and CA1 hippocampal subfields with select hearing and cognition measures. Ultimately, this work may provide a framework to assess the efficacy of hearing intervention and cognitive intervention in those with hearing loss.

